# Clinical efficacy of Yingliu mixture combined with metimazole for treating diffuse goitre with hyperthyroidism and its impact on related cytokines

**DOI:** 10.1080/13880209.2016.1260595

**Published:** 2016-12-07

**Authors:** Hua Yang, Yilei Cong, Tengfei Wu, Hong Tang, Muhao Ma, Juanhua Zeng, Wenya Zhang, Zhen Lian, Xinyu Yang

**Affiliations:** Department of Endocrinology, Affiliated Longhua Hospital of Shanghai University of Traditional Chinese Medicine, Shanghai, China

**Keywords:** Graves’ disease, cytokines, traditional Chinese medicine

## Abstract

**Context:** Yingliu mixture was developed in 1990s by Affiliated Longhua Hospital of Shanghai University of Traditional Chinese Medicine, for treating diffuse goitre with hyperthyroidism (Graves’ disease, GD). Former studies have shown Yingliu mixture combined with methimazole (Y-M) can effectively improve thyroid function and decrease thyrotropin-receptor antibody level. Furthermore, we researched its impact on related cytokines to prove that Y-M improve patients’ immunity status.

**Objective:** To observe the clinical efficacy of Y-M for treating GD.

**Methods:** A total of 120 GD patients were randomly divided into two groups, the treatment and the control groups (*n* = 60). The treatment group’s patients were treated with Y-M. The control group’s patients were treated with methimazole alone. Yingliu mixture was orally administered, 25 mL three times daily. Methimazole was administered at 5–25 mg/day. After 12 weeks of the treatment, the cytokines, antibodies related to thyroid function, and Chinese medical syndromes were evaluated.

**Results:** After the treatment, the free triiodothyronine and thyroxine levels in both groups decreased. The thyroid-stimulating hormone level increased in the treatment group. The thyrotropin-receptor antibody levels and TNF-α levels decreased in both groups. In the control group, IL-6 and IFN-γ levels were lower than that before the treatment. In the treatment group, CD4^+^ and CD25^+^ levels were higher than pretreatment levels, but IL-10 levels were reduced. Clinical symptoms: the total CMS scores for both groups decreased.

**Conclusions:** The Y-M combination can improve thyroid function, and decrease autoantibodies, cytokines, and clinical symptoms, so its efficacy may surpass that of methimazole alone.

## Introduction

Graves’ disease (GD) is the most common type of hyperthyroidism and features a diffuse goitre (Yang et al. 2002; Teng et al. 2006). GD accounts for 88% of all patients with hyperthyroidism and although its prevalence is approximately 1–2%, a gradual increase has occurred in recent years. GD is an autoimmune disease, and untreated hyperthyroidism could cause damage to multiple systems including the cardiovascular system (hyperthyroid heart disease), and the blood, nervous, digestive, and reproductive systems, thus seriously affecting the quality of life of patients.

Immune factors are the core problem in GD. Patients with GD exhibit abnormal cytokine expression compared with healthy individuals, so cytokines participate in the disease process and in the development of GD. GD is a an autoimmune disease based on the humoural immune response, and possibly results from an imbalance in the regulation of a variety of immune cells during its pathogenesis, thereby stimulating the production of thyrotropin-receptor antibodies (TRAb) that finally leads to GD (Weetman 2000). In animal studies (Wakelkamp et al. 2000), mice with a knockout of regulatory T (Treg) cells had reduced inhibition of Th cells, and the increased production of TRAb consequently became the main cause of GD in this model. Increasing the activity of Tregs could inhibit autoimmunity, and is a promising therapeutic strategy for treating GD. Currently, Western medical treatments (^131^I, anti-thyroid drugs, and surgery) mainly address the symptoms (Cooper 2005; Ross 2011) and have some advantages, as well as disadvantages (Bahn et al. 2011) such as the long duration of drug treatment and high recurrence rate (Daukšienė et al. 2013; Yoshihara et al. 2014). Traditional Chinese medicines have made certain advances by regulating immunity in those with GD, and our custom Yingliu mixture has been based on many years of clinical observations by our department, with confirmed efficacy towards GD owing to its characteristic of supplementing Qi and nourishing Yin. This study has explored its clinical efficacy, with a preliminarily investigation of the mechanisms to enhance the theoretical basis.

## Materials and methods

### Patients

A total of 120 GD patients were selected from outpatients or inpatients that met the inclusion criteria and were treated at the Department of Endocrinology, Longhua Hospital, from September 2012 to July 2015. They were randomly divided into treatment [treated with Yingliu mixture (Y-M) combined with methimazole] and control groups (only treated with methimazole), 60 patients in each group. This study was conducted in accordance with the declaration of Helsinki. This study was conducted with approval from the Ethics Committee of Shanghai University of Traditional Chinese medicine. Written informed consent was obtained from all participants.

### Inclusion criteria

Patients were included who (1) met the diagnostic criteria for GD (with reference to the Chinese treatment guidelines for thyroid diseases, Chinese Society of Endocrinology 2007) (Yin et al. 2014); (2) met the subtyping for Qi-Yin Deficiency combined with Phlegm syndromes in TCM (with reference to the ‘Clinical research guidelines of new Traditional Chinese Medicines (Trial)’); (3) were aged between 18 and 70 years (including 18 and 70 year olds), but without a gender restriction; and (4) provided informed consent and could cooperate with regular follow-up.

### Exclusion criteria

Patients were excluded who (1) did not meet the above diagnostic criteria; (2) were without goitre; (3) were pregnant or lactating; (4) had been diagnosed with leucemia, aplastic anaemia, leucopenia (white blood cell count [WBC] < 3.0 × 10^9^/L), neutropenia (absolute neutrophil count <1.5 × 10^9^/L), primary diseases of the cardiocerebral vessels, liver, or kidney, or mental illness; (5) were experiencing a thyroid crisis; (6) had significant goitre growth that compressed adjacent organs; (7) had various types of thyroiditis without hyperthyroidism; (8) had an allergic constitution or were allergic to multiple drugs; (9) had taken anti-thyroid drugs within the past 3 months; (10) had levels of alanine aminotransferase more than double the normal upper limit before the treatment.

### Criteria for discontinuing patient participation

Patients were discontinued who (1) were found not to meet the selection criteria during the study; (2) were without adverse drug events during the study, but had to interrupt the medication for other reasons (such as other illnesses); (3) abandoned their trial participation because they considered the drugs ineffective; (4) experienced serious adverse events during the treatment; (5) could not proceed in accordance with the treatment program, with the patient or family requesting that the medication be withdrawn; (6) experienced an exacerbation of the disease, or other syndromes developed that would affect the observation of this trial, and the patient was stopped from participating according to the judgment of the practitioners; (7) were allergic to the test drugs.

### Abandonment criteria

The trial would be abandoned if (1) serious adverse reactions occurred; (2) there appeared to be no effect, or liver damage (ALT ≥ twice the normal upper limit), allergies developed, WBC <2.5 × 10^9^/L, or absolute neutrophil count <1.5 × 10^9^/L.

### Treatment

Basic treatment: the two groups were told to avoid iodine-containing foods, have a relaxed mental state, and quit alcohol and smoking. Each course of observations was set at 12 weeks.

Treatment group: Yingliu mixture combined with methimazole (Saizhi); the Yingliu mixture was prepared by staff in the preparation room at the Longhua Hospital affiliated with the Shanghai University of Traditional Chinese Medicine, using drug approval No: Hu Pharmacy Z05170226. It was the mixed extracts of decoction from TCM including *Radix astragali* Leguminosae, *Radix scrophulariae* Scrophulariaceae, *Radix ophiopogonis, Ophiopogon japonicus*, *Rhizoma anemarrhenae* Liliaceae, *Fructus forsythiae* Oleaceae, *Spica prunellae* Labiatae, *Concha ostreae* Ostreidae, *Bulbus fritillariae thunbergii* Liliaceae, *Smilax china* Linn Liliaceae, *Semen sinapis* Brassicaceae, *Semen impatientis* Balsaminaceae. Dosage: oral administration, 25 mL three times daily. Methimazole (Saizhi) was administered at 5–25 mg/day, with the specific amount decided according to the specific disease conditions (Merck Co., Darmstadt, Germany, Specification: 10 mg ×50 tablets; state medical Approval No: H20070289).

Control group: methimazole (Saizhi) 5–25 mg/day, with the specific amount decided according to the specific disease conditions (Merck Co., Specification: 10 mg ×50 tablets; state medical Approval No: H20070289).

Indications for co-medication: Patients in both groups with a heart rate >100 beats/min and no history of asthma could be co-administered metoprolol tartrate tablets (Betaloc), dosage 23.75–47.5 mg four times daily; those with a history of asthma could be administered diltiazem hydrochloride tablets, 30 mg three times daily; patients with ALT exceeding the normal upper limit could also be administered diammonium glycyrrhizinate enteric-coated capsules (Chiatai Tianqing Pharma, Jiangsu Province, China), at a dosage of 50–100 mg three times daily; patients with blood leucocytes <3.5 × 10^9^/L could be administered leucogen tablets (Leucogen), 20 mg two or three times daily.

### General observations

Data included name, sex, age, disease duration, blood pressure, respiration, heart rate, and body temperature.

### Safety observations

(1) Routine blood (LH750 Blood Cell Analyzer, Beckman, Carlsbad, CA) and liver function tests (DB-001automatic biochemical analyzer, Roche, Basel, Switzerland) were performed: once before the commencement of the treatment, 2 weeks after the treatment commenced, 6 weeks after the treatment commenced, and at the end of the treatment period.

(2) Renal function testing (DB-001 automatic biochemical analyzer, Roche, Basel, Switzerland) was performed once before the commencement of the treatment and at the end of the treatment.

### Efficacy indicators

The main symptoms of disease included fatigue and acratia, dry mouth or thirst, heat intolerance, sweating, chest tightness, excessive phlegm, emotional upsets, irritability, loss of sleep and dreaminess, eating more while still losing weight, dry eyes, blurred vision, petechia on face, as well as changes to the tongue and pulse. The presence or absence of the above symptoms was recorded once before the commencement of the treatment, and again after 2, 4, 6, 8, 10, and 12 weeks of the treatment, respectively. The signs of disease including changes in body weight, heart rate, and goitre were recorded once before the treatment, then after 2, 4, 6, 8, 10, and 12 weeks of the treatment, respectively. The scoring criteria for TCM syndromes were according to the Clinical guideline of new drugs for Traditional Chinese Medicine (2002 edition).

The analytical test indexes including free triiodothyronine (FT3), free thyroxine (FT), thyroid-stimulating hormone (TSH), TRAb, anti-thyroid-peroxidase antibody (TPOAb), and anti-thyroglobulin antibody (TGAb) were measured according to reported methods (Gu et al. 2016) once before the commencement of the treatment, 6 weeks after the treatment commenced, and at the end of the treatment, respectively. The routine blood tests were performed once before the commencement of the treatment, 2 weeks after the treatment commenced, 6 weeks after the treatment commenced, and at the end of the treatment, respectively. CD4^+^/CD25^+^, IL-6, IL-10, TNF-α, and interferon-γ were assayed once before and once after the treatment according to the method reported by Dieckmann et al (2002). In addition, ECG (CardioEX ECG, Huatai Co., Shanghai, China) and ultrasound imaging of the thyroid (Siemens 2000, Siemens Co., Berlin, Germany) were conducted once before and once after the treatment, respectively.

### Statistical analysis

SPSS17.0 software was used for the statistical analysis. If normally distributed, the measurement data were then expressed as mean ± standard deviation, otherwise, as the median (interquartile range) for the statistical description. The measurement data were compared using the independent-sample *t*-test or non-parametric test, as well as ANOVA for the repeated-measures data. The counting data were analyzed using the chi-square test, and ranked data were analyzed using the rank sum test, with *p* < 0.05 considered statistically significant.

## Results

### General information

This study included a total of 120 GD patients with Qi-Yin Deficiency, and the data for sex, age, disease duration, and main clinical indicators showed no statistically significant differences between the two groups, so they were comparable (Table 1).

**Table 1.** Comparison of baseline data between the two groups.

Item	Control group	Treatment group	*p*
N (cases)	65	65	–
M (cases)	17	8	0.44
F (cases)	48	57	
Age (years)	40.32 ± 14.192	42.92 ± 13.467	0.81
FT3 (pmol/l)	14.90 (20.91)	11.88 (17.12)	>0.05
FT4 (pmol/l)	38.08 (53.37)	34.98 (51.89)	>0.05
TSH (mIU/L)	0.005 (0.004)	0.005 (0.0035)	>0.05
TGAb (IU/ML)	87.35 (261.44)	246.7 (493.76)	>0.05
TPOAb (IU/ML)	103.0 (310.0)	129.4 (316.67)	>0.05
TRAb (mIU/ML)	8.83 (20.1)	8.48 (20.06)	>0.05
Symptom score	33.51 ± 7.115	37.06 ± 6.619	>0.05
CD4^+^/CD25^+^ (%)	9.3 (4.95)	6.7 (5.45)	>0.05
IL-10 (pg/ml)	5.0 (0)	5.0 (0.6)	>0.05
IL-6 (pg/ml)	2.2 (1.2)	2.0 (0.6)	>0.05
TNF-α (pg/ml)	6.8 (6.1)	8.76 (7.15)	>0.05
IFN-γ (pg/ml)	7.0 (4.95)	6.3 (5.45)	>0.05

### Thyroid function

Comparisons were performed using data obtained before the commencement of the treatment and 6 weeks after the treatment commenced. Intragroup comparison: compared with the pretreatment data, FT3 and FT4 levels in both groups were decreased (*p* < 0.001), but TSH levels in both groups showed no statistically significant differences, indicating that thyroid function in both groups was effectively improved. Intergroup comparison: FT3, FT4, and TSH in both groups showed no statistically significant differences, indicating no difference in efficacy between the two groups after 6 weeks of the treatment.

Comparisons were performed of data obtained before the commencement of the treatment and 12 weeks after the treatment commenced. Intragroup comparison: compared with the pretreatment data, FT3 and FT4 levels in both groups were statistically decreased (*p* < 0.001), but TSH levels in the treatment group were significantly increased (*p* < 0.05), indicating that both groups could effectively reduce FT3 and FT4 levels, and the treatment group could also effectively increase TSH levels. Intergroup comparison: FT3, FT4, and TSH levels showed no statistically significant differences between the two groups after 12 weeks of the treatment.

### Thyroid autoantibodies

Comparisons were performed using the data obtained before the commencement of the treatment and 6 weeks after the treatment commenced. Intragroup comparison: compared with the pretreatment data, TGAb, TPOAb, and TRAb levels for both groups showed no statistically significant differences (*p* > 0.05). Intergroup comparison: TGAb, TPOAb, and TRAb levels in both groups showed no statistically significant differences (*p* > 0.05), indicating no difference in efficacy between the two groups after 6 weeks of the treatment.

Comparisons were performed of data obtained before the treatment and 12 weeks after the treatment commenced. Intragroup comparison: compared with the pretreatment data, TGAb and TPOAb in both groups had decreased but the difference was not statistically significant (*p* > 0.05). TRAb levels in both groups were decreased compared with those after 6 weeks of the treatment (*p* < 0.05 and *p* < 0.001, for the control and the treatment groups, respectively), and were also significantly decreased compared with levels measured before the treatment (*p* < 0.001), indicating that both the treatments could decrease TRAb levels, and the TRAb decrease in the treatment group occurred earlier and lasted longer. Intergroup comparison: TGAb, TPOAb, and TRAb showed no significant differences between groups. However, the decreasing trend for TRAb levels was greater in the treatment group than in the control group (Table 2).

**Table 2.** Comparison of thyroid functions and antibodies before and after the treatment.

	Control group			Treatment group		
Item	Before	6-week after	12-week after	Before	6-week after	12-week after
FT3 (pmol/l)	17.59 ± 12.15	9.54 ± 6.99**	8.11 ± 6.0**^▴▴^	16.85 ± 13.08	8.85 ± 6.27**	6.98 ± 4.45**^▴▴^
FT4 (pmol/l)	46.21 ± 28.54	25.88 ± 17.53**	22.74 ± 16.18**^▴▴^	46.50 ± 30.46	26.90 ± 16.73**	20.71 ± 10.61**^▴▴^
TSH (mIU/L)	0.86 ± 5.83	1.79 ± 7.67	1.76 ± 5.31	0.77 ± 3.65	1.15 ± 2.75	1.79 ± 5.47*
TGAb (IU/ML)	276.76 ± 579.16	288.43 ± 541.47	243.74 ± 468.38	473.10 ± 763.72	443.70 ± 759.33	453.51 ± 863.43
TPOAb (IU/ML)	192.24 ± 198.43	188.53 ± 199.17	194.45 ± 199.44	196.18 ± 191.98	201.65 ± 231.73	169.20 ± 178.16
TRAb (mIU/ML)	23.12 ± 73.73	13.73 ± 12.24	11.48 ± 11.78^▴^	13.53 ± 12.76	11.89 ± 12.26	9.15 ± 10.82**^▴▴^

Intragroup comparison with that before the treatment *p < 0.05, **p < 0.001, intragroup comparison with that 6-week after treatment ^▴^*p* < 0.05, ^▴▴^*p < *0.001, and no significant difference was detected between groups at the same time points.

### Thyroid-related cytokines

Comparisons were made of CD4^+^ CD25^+^, IL-6, IL-10, TNF-α, and IFN-γ levels before and after the treatment. TNF-α levels in both groups were lower than those measured before the treatment (*p* < 0.05 and *p* < 0.001 for the control and the treatment groups, respectively). IL-6 and IFN-γ levels in the control group were lower than those before the treatment (*p* < 0.05 and *p* < 0.01, respectively). In the treatment group, CD4^+^ CD25^+^ levels were increased after the treatment (*p* < 0.05), but IL-10 was reduced (*p* < 0.05). There was no significant difference in the cytokine levels between the two groups before and after the treatment (Table 3).

**Table 3.** Comparison of CD4^+^ CD25^+^, IL-6 and 10, TNF-α, and IFN-γ between the two groups before and 12 weeks after the treatment (M (QR)).

	CD4^+^/CD25^+^		IL-10		IL-6		TNF-α		IFN-γ	
Groups	Before	After	Before	After	Before	After	Before	After	Before	After
Control	9.3 (4.95)	9.1 (4.6)	5.0 (0.0)	5.0 (0.0)	2.2 (1.2)	2.0 (0.4)*	6.8 (6.1)	4.4 (5.88)**	7.0 (4.95)	6.0 (4.55)*
Treatment	6.7 (5.45)	8.1 (4.45)*	5.0 (0.6)	5.0 (0)*	2.2 (0.6)	2.0 (0.3)	8.76 (7.15)	5.5 (5.8)*	6.3 (5.45)	6.9 (6.4)

Compared with that before the treatment *p < 0.05, **p < 0.01, and no significant difference was detected between groups at the same time points.

### Total scores for TCM syndromes

ANOVA of repeated measurements showed statistically significant differences at different time points within each group (*p* < 0.001), indicating that the total scores for TCM Syndromes were improved at different time points in both groups. However, the intergroup difference was not statistically significant, therefore, it could not be determined whether the decreasing trends of the two groups were different (Table 4).

**Table 4.** Comparison of total score of TCM syndromes at different time points between the two groups (*x* ± s).

Group	Before	6-week after	12-week after
Control	33.71 ± 6.789	26.26 ± 6.109**	21.29 ± 6.578**^▴▴^
Treatment	35.00 ± 6.745	25.46 ± 5.596**	18.22 ± 6.496**^▴▴^

Intragroup comparison with that before the treatment *p < 0.05, **p < 0.001, intragroup comparison with that 6-week after the treatment ^▴^*p < *0.05, ^▴▴^*p < *0.001, and no significant difference was detected between groups at the same time points.

### Improvements in main symptoms and signs

Effective improvements in goitre were seen after 12 weeks of the treatment in 28 patients from the treatment group and 9 patients from the control group; effective improvements in fatigue and acratia were seen in 61 patients from the treatment group and 54 patients from the control group; effective improvements in shortness of breath were seen in 56 patients from the treatment group and 48 patients from the control group; effective improvements in excessive thirst were seen in 54 patients from the treatment group and 42 patients from the control group. The chi-square test showed that the effectiveness rates for improving goitre and thirst showed statistically significant differences between the two groups (*p* < 0.05), indicating that the efficacy in the treatment group was better than that in the control group (Table 5).

**Table 5.** Comparison of total efficiencies in improving goitre, shortness of breath, and thirst between the two groups.

	Goitre		Fatigue and acratia		Breath shortness		Dry mouth and thirst	
Group	Ineffective	effective	Ineffective	effective	Ineffective	effective	Ineffective	effective
Control	56	9	11	54	17	48	23	42
Treatment	37	28^▴^	4	61	8	56	11	54^▴^

Compared with the treatment group, ^▴^*p* < 0.05.

The comparison of the clinical TCM scores between the two groups showed that the treatment group exhibited better efficacy. After the 12-week of treatment, the treatment group had 0 case of clinical cure, 5 cases showing significant improvement, 52 cases of effective, and 8 cases of ineffective, and the total effectiveness rate was 87.7%; the control group had 0 case of clinical cure, 6 cases of significantly effective, 28 cases of effective, and 31 cases of ineffective, and the total effectiveness rate was 52.3%. Non-parametric testing showed that the difference in clinical efficacy between the two groups was statistically significant (*p* < 0.05), indicating that the efficacy in the treatment group (Y-M) was better than that in the control group (M alone) (Table 6).

**Table 6.** Efficacies of main TCM syndromes in the two groups.

Group	*n*	Cure	Significantly effective	Effective	Ineffective	Total effective rate (%)
Control	65	0	6	28	31	52.3
Treatment	65	0	5	52	8	87.7^▴^

Compared with the control group ^▴^*p <* 0.05.

## Discussion

GD can occur at any age, and is more common in females than in males. Currently, there is no effective treatment method. Immune factors are the core problem in GD, among which cytokines play extensive roles in regulating the immune function of the body, as well as having an important influence on the function of thyroid follicular epithelial cells (Stefan et al. 2014). It has already been confirmed that immune function disorders are present in GD, and an imbalance between the T-helper cell 1 (Th1) and T-helper cell 2 (Th2) populations plays a key role in the occurrence and development of GD (Wakelkamp et al. 2000). Th1 cells mainly secrete IL-2 and IFN-γ (Yao et al. 2014), while Th2 cells mainly secrete IL-6 and IL-10 (He et al. 2014). Among these cytokines, IFN-γ could induce the thyroid cells to express HLA-I molecules and HLA-II molecules, and could also inhibit the expression of thyroid molecules such as thyroglobulin, TPO, and TSH, thus leading to decreased production of thyroglobulin and TPO and decreased absorption of iodine. On the one hand, IFN-γ could enhance thyroid antigenicity, and on the other hand, it could inhibit cell differentiation (Kennedy & Jones 1991). IFN-γ and IL-6 are both highly expressed in the early stages of GD, so they might be involved in disease pathogenesis, and could be used as indexes for evaluating immune dysfunction (Stefan et al. 2014). Esfahanian et al. (2013) found that the serum levels of IL-2 and IL-10 in GD patients were significantly higher than those in the control group, suggesting that patients with GD mainly exhibit humoral immunity, and IL-10 could be a novel drug target. Of course, in addition to Th1/Th2 cells, Th17 cells also secrete IL-17 and other cytokines, and play an important role in the pathogenesis of autoimmune diseases (Bedoya et al. 2013; Rodrigues-Díez et al. 2014). TRAb is a human-specific antibody, and is the main direct cause of GD at its onset. TRAb has an important function in the pathogenesis of hyperthyroidism; firstly, 70–95% of the patients with newly diagnosed GD are positive for TRAb; secondly, TRAb could also predict the recurrence of GD, and the sensitivity and specificity of TRAb for predicting recurrence after antithyroid drug therapies were both more than 50%. During the treatment, the decrease in the TRAb titre could be seen as a sign of GD improvement; therefore, regularly monitoring TRAb could guide clinicians in judging whether the disease was remitted, and provide a reference for reducing or withdrawing the medication. An absence of TRAb in the late treatment of GD patients could reflect remission of the disease (Wallaschofski et al. 2002). TPOAb and TGAb are autoimmune antibodies specific to thyroid diseases; titres could be increased in early GD, and with remission of the disease, the titres might decrease (Colobran et al. 2011; Latrofa et al. 2014). This study observed changing trends in different cytokines during the test, consistent with the results of previous studies to some extent. However, the overall changes were not significant, which might mainly reflect the small sample size; therefore, our future studies might need to appropriately increase the sample size. Moreover, not all the GD patients exhibited abnormal cytokines, so patients with abnormal cytokine profiles could be targeted and screened to study the changes. In addition, the relevance of different cytokines to the GD immune process should also be studied further.

We believe that TCM is effective at improving immunity. Compared with the results for TNF-α and IL-6, TCM could much more effectively elevate CD4^+^ CD25^+^, decrease IL-10, and have early and longer effects on TRAb levels.

From the viewpoint of Chinese medicine, this study used one TCM prescription, Yingliu mixture. This exhibits its main potency by supplementing Qi and nourishing Yin. We aimed to observe whether the combination of Yingliu mixture with the Western drug, methimazole, could better regulate the immune function of patients with GD. The results suggest that the combination of Yingliu mixture and methimazole could effectively reduce TRAb levels, thus achieving a rapid improvement in the symptoms and shortening the treatment course. In Chinese medicine, GD belongs to the category of Qi goitre, and is mainly caused by a long-term Yin deficiency-induced gradual Qi-Yin deficiency. Upset emotions, depression, and anxiety in patients are symptoms of asthenia in origin (Qi-Yin deficiency) and asthenia in superficiality (Qi stagnation, phlegm coagulation, and blood stasis). This TCM prescription mainly uses *Radix astragali* as the sovereign drug, aiming to nourish the middle Qi in the spleen and stomach; uses *Radix scrophulariae*, *Radix ophiopogonis*, and *Rhizoma anemarrhenae* as the minister drug to cleanse heat, nourish Yin, and relieve anxiety; uses fructus forsythiae and spica prunellae to clear heat and purge fire, as well as concha ostreae, bulbus fritillariae thunbergii, smilax China, and semen sinapis to dissipate phlegm, resolve masses, and soften hard masses, and semen impatientis to disintegrate blood stasis and soften hard masses. The combination of these TCMs has the role of supplementing Qi and nourishing Yin, clearing heat and dissipating phlegm, and activating blood and resolving masses, thus differentially improving the symptoms of Qi-Yin deficiency in patients. According to modern research, the TCMs in this prescription each have different functions in immunity; for example, *Radix astragali* could promote the function of immune cells, and reduce or eliminate the activity of inhibitory lymphocytes (Wei et al. 2004); *Radix scrophulariae* could better restore immune function in Yin-deficient mice (Bermejo Benito et al. 2000); *Radix ophiopogonis* could improve cell viability and regulate the function of immune cells (Xiong et al. 1958). This study observed that after the treatment, the clinical symptoms in the treatment group were significantly improved. Although no significant difference was observed among cytokine levels before and after the treatment, a declining trend for TRAb and an increasing trend for CD4^+^ CD25^+^ were more obvious in the treatment group than in the control group. The current results might be related to the short observation and the treatment period, which could be further extended to better evaluate the efficacy.

Yingliu mixture combined with methimazole could reduce TNF-α and IL-10, increase CD4^+^ CD25^+^, improve thyroid function, reduce thyroid autoantibodies, and improve the clinical symptoms in patients with GD, and the efficacy was shown to be better than that obtained by the use of methimazole alone.
